# The effect of resource availability on interspecific competition between a native and an invasive ant

**DOI:** 10.1098/rstb.2021.0146

**Published:** 2022-05-23

**Authors:** Kevin Neumann, Noa Pinter-Wollman

**Affiliations:** Department of Ecology and Evolutionary Biology, University of California, Los Angeles, 621 Charles E. Young Drive South, Los Angeles, CA 90095, USA

**Keywords:** collective behaviour, argentine ant, *Tapinoma sessile*, aggression, exploration

## Abstract

Interspecific competition influences the composition of ecological communities. Species may differ in their needs for different resources, therefore resource availability may determine the outcome of interspecific interactions. Species often compete over food, shelter or both. When more than one resource is limited, different species may prioritize different resources. To determine the impact of resource availability on the competitive relationship between an invasive and a native species, we examined interactions between groups of the invasive Argentine ant (*Linepithema humile*) and the native odorous ant (*Tapinoma sessile*) over (1) food, (2) shelter or (3) both simultaneously. We further examined the mechanisms underlying the competitive relationship, asking whether aggressive interactions, exploratory behaviour or the order of arrival at a resource explained resource use. Shelter was preferred by both species when no competitors were present. In a competitive setting, *L. humile* groups controlled shelter through aggressive displacement but lost control over food due to investment of workers in the control of shelter. Thus, there are tradeoffs when competing over multiple resources and aggressive interactions allow invasive species to displace native species from a preferred resource.

This article is part of the theme issue ‘Intergroup conflict across taxa’.

## 1. Introduction

Interspecific competition is pervasive across ecological systems, influencing species range and distribution. Heterospecific competitors can impact the survival of individuals from other species by decreasing fecundity [1] or limiting access to resources [2,3]. Competition may influence the spatial [4,5] or temporal [6,7] partitioning of resources, and resource specialization leading to coexistence. Animals may displace competitors from resources [8–10], or avoid encounters with competitors [11,12].

Animals often compete over multiple resources such as food and shelter. Interspecific interactions can limit access to both food [8,13] and shelter [14]. For example, some social insects avoid interactions near food [15,16] and heterospecifics occupying high-quality nests may lead some social insects to lower-quality nests during nest relocation [17]. Studies of interspecific competition often examine interactions over a single type of resource, however, animals require more than one type of resource and may need to prioritize which resource to compete over when their competitive effort is limited. When social species interact, individuals can be allocated to different resources to facilitate the control of multiple resources simultaneously. Large groups often outcompete smaller ones in interspecific competitions [18–20]. So, when group members are spread across multiple resources, the ability to control all resources may be compromised for small groups that might not be able to defend multiple resources or even a single resource. Here, we investigate how interspecific competition between social animals is influenced by the presence of multiple types of resources compared to competition over a single resource.

Different behavioural mechanisms may underlie animals' ability to gain and control resources. Direct aggressive interactions displace competitors and reduce the likelihood of future competition [21]. Exploratory behaviour can lead to the discovery of resources [22], and resource discovery often results in resource dominance [23,24]. Still, aggressive individuals that arrive at a resource late can outcompete the individuals who discovered the resource [25]. Although aggression and exploration can play crucial roles in interspecific encounters, it is unclear how they facilitate interspecific interactions over multiple resources. Social insects provide ample opportunities for examining these open questions because of the wide range of inter-specific competition they exhibit [26].

The Argentine ant (*Linepithema humile*) is an invasive species throughout the world [27] that often displaces native ant species [28,29], including the odorous house ant (*Tapinoma sessile*), which is native to North America. Both *L. humile* and *T. sessile* live in large polygynous and polydomous colonies [30], nest in underground cavities, have monomorphic workers of similar size and exhibit individual variation in aggression [31–33] and exploration [34,35]. In one-on-one interactions, *T. sessile* generally outcompete *L. humile*, but when entire colonies compete, *L. humile* is typically the dominant species [31]. The outcome of competition between small groups, in which the number of individuals that can be allocated to a resource is limited, has not been examined thus far. Interactions among small groups of *L. humile* and *T. sessile* are of particular interest because both species expand their range via budding when a propagule—a small group of workers and at least one queen—leaves the nest to found a new colony [36,37]. Examining the interspecific competition among small groups of *L. humile* and *T. sessile* may reveal limits on the dispersal and spread of a highly invasive species.

Here, we examine how interspecific competition between small groups of *L. humile* and *T. sessile* workers is affected by the availability of multiple resources—food and shelter. We hypothesize that (1) interspecific competition will influence survival and resource use and predict that the ecologically dominant species (*L. humile*) will have higher survival and that it will control both resources, while the less dominant species (*T. sessile*) will have lower survival and be excluded from both resources; (2) interspecific competition over a single resource differs from interspecific competition over multiple resources simultaneously; we predict that *L. humile* will successfully dominate a single resource, but will only dominate one resource when two resources are provided because of an allocation-tradeoff; and (3) interspecific competition is driven by aggression, exploration or resource discovery. Based on past work on the aggression of *L. humile* toward other ant species [38,39], we predict that aggression will be the predominant mechanism driving interspecific competition.

## 2. Methods

### (a) Collection and maintenance of ants

Throughout the study, between January and June 2018, we collected approximately 3000 *L. humile* workers from foraging trails at the UCLA Mildred E. Mathias Botanical Gardens, CA, USA, where *L. humile* are the predominant ant species present (K. Neumann 2018, personal observations). Ants were collected with an aspirator every 2–3 weeks in batches of a few hundred individuals each time to avoid maintaining ants in the laboratory for long. Ants were used in an experiment within two to three weeks of collection. While different foraging trails at the botanical gardens were used for collection, they were all within 200 m of each other so they can be considered to all belong to a single effective colony [40]. We housed workers in a 5200 ml container in the laboratory and provided them with nests, water and sugar water *ad libitum*. Laboratory temperature was kept constant at 23°C throughout the experiments and lights were set to a 12 : 12 D : L cycle. Similarly, throughout the study we collected approximately 3000 *T. sessile* workers from a persistent foraging trail at a residential area in Venice, Los Angeles, CA, USA, where no *L. humile* are found and where *T. sessile* is the predominant ant species (N. Pinter-Wollman 2018, personal observations). Again, these workers were collected in small batches every few weeks to reduce the time they spent in the laboratory. *Tapinoma sessile* workers were kept in identical conditions to those of *L. humile* workers. Although foraging trails represent only a fraction of the entire colony, workers along these foraging trails vary in their individual behaviour [33,35,41]. We did not include queens or brood in the experimental groups because we could not obtain a large enough number of brood and queens when collecting ants without destroying the nests. It is possible that ants might behave differently in the presence of queens and brood; however, workers on foraging trails (without queens and brood) are the most likely to encounter other ant species and participate in interspecific competition.

### (b) Interspecific competition assays

**Figure 1.**
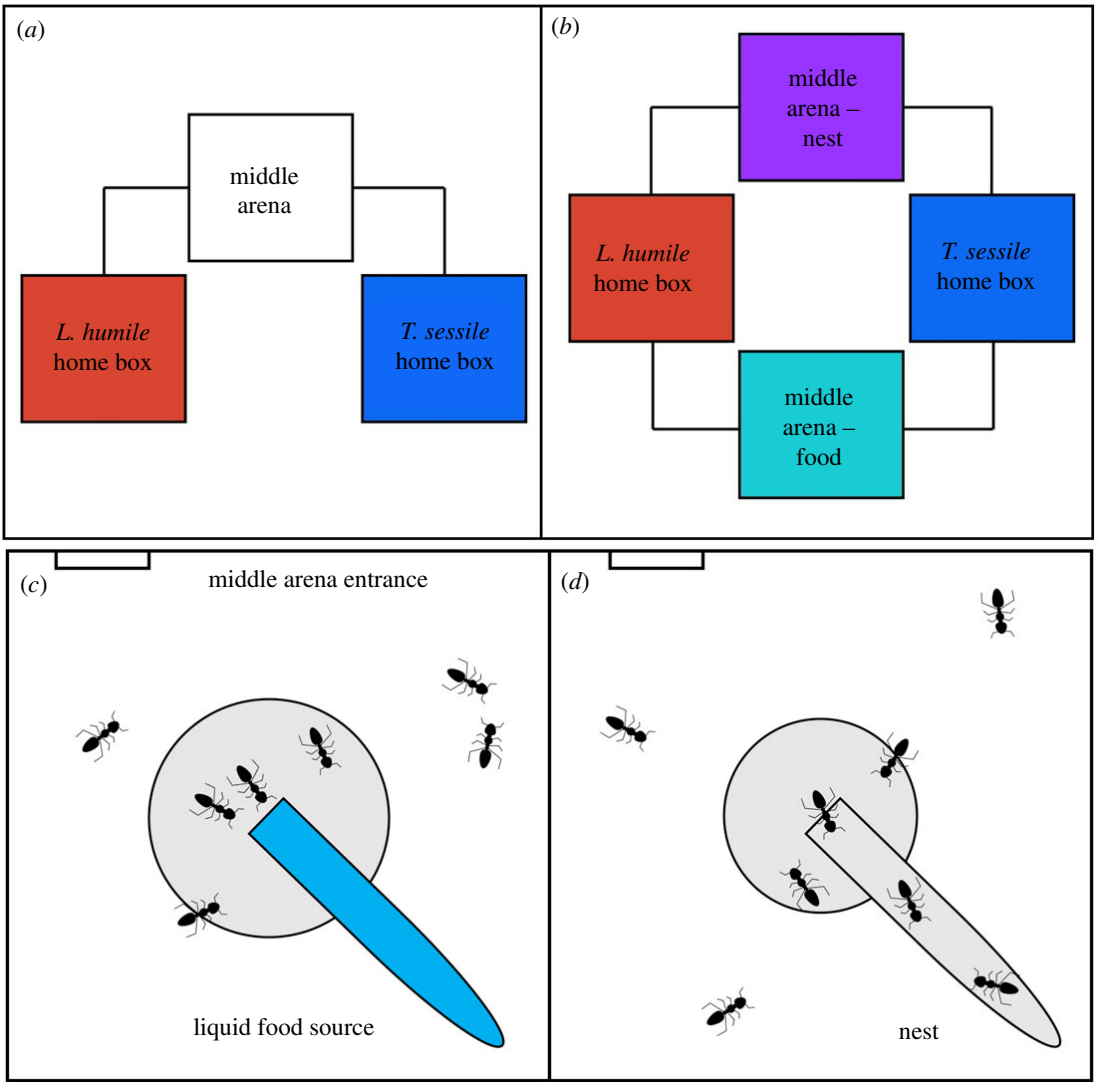
Study apparatus. (*a*) Schematic of the experimental apparatus with one resource and (*b*) apparatus with two resources. Each square represents a plastic box (710 ml, 12 × 12 cm) and the connecting lines represent plastic tubes, each 20 cm long with an opening diameter of 0.75 cm (photo of the one resource apparatus can be found in electronic supplementary material, figure S1). To determine resource use we counted the number of ants within the gray area near food (*c*) or nest (*d*). Both resources were provided in a glass vial. Food (sugar water) filled the vial (*c*), and the nest was covered in tinfoil (not shown here for visibility) and contained a wet cotton ball at the bottom for humidity. Because the ants could enter the vial when it was a nest (*d*) but could not enter it when it was full of food (*c*), the circles indicating proximity to the resource are of slightly different sizes (nest: radius = 3 cm; food: radius = 3.4 cm) to account for the use of the area inside the vial itself. Ants are for illustration purpose only and their size is not to scale. (Online version in colour.)

We examined the competitive interactions between groups of *L. humile* and *T. sessile* workers in two study apparatus. To examine interspecific competition over a single resource, we connected three plastic boxes (710 ml, 12 × 12 cm each) using plastic tubes of approximately 20 cm in length, and an inner diameter of 0.75 cm (figure 1*a,b* and electronic supplementary material, figure S1). In one box we placed 50 *L. humile* workers and in a second we placed 50 *T. sessile* workers—both referred to as the ‘home boxes'. In the third box, which was between the two home boxes and we referred to as the ‘middle arena’ (figure 1*a*, electronic supplementary material, figure S1), we placed a resource (food or nest). Groups of 50 workers were selected to represent a dispersing propagule [36,37]. To test competition over multiple resources simultaneously, we used an apparatus in which four plastic boxes were connected by plastic tubes, creating two ‘middle arenas’ (figure 1*b*). We introduced 50 workers of each species into the two home boxes and placed food into one middle arena and a nest into the other middle arena (figure 1*c,d*).

To allow ants to acclimate to the study apparatus, after placing the ants in the home boxes we blocked the home boxes from the middle arena(s) for 8 h using cotton balls, which plugged the plastic tubes leading into the middle arena(s). During the acclimation period, ants were provided with a nest and sugar water in their home boxes. Each nest was made of a glass vial (10 cm) with a damp cotton ball inside and a tinfoil cover to create a dark humid space. Sugar water (20% concentration) was provided in a glass vial plugged with a cotton ball to prevent the sugar water from spilling and to allow the ants access to the sugar water that wicked through the cotton. The amount of water and sugar water in the vials was substantial and did not require replacement during the acclimation period or during the experiments.

To test competition over resources (nest, food, or both) we removed either the nest or food or both from the home boxes of both species. When examining competition over food we removed the food entirely and when examining competition over a nest we only removed the tinfoil covering the home nest to minimize the amount of disruption during the experimental manipulation. When examining competition over both food and nest simultaneously, we removed both the food and the tinfoil cover from the home boxes. We performed eight replicates for each type of competition over (1) only food, (2) only nest and (3) both food and nest simultaneously. Each replicate for each trial was conducted with a different group of 50 workers; thus, we used 24 different groups of 50 workers for each species. To control for interspecific competition, and to determine resource use without a heterospecific, we repeated these three assays four more times for each species without the heterospecific present at the end of the study, after completing the treatment experiments. Again, each control trial was conducted with a different group of 50 workers, thus 12 different groups of 50 workers from each species were used in the control trials. Test apparatus was thoroughly cleaned with ethanol between trials.

### (c) Quantifying resource use

To quantify the use of resources in the arena(s), we manually counted the number of ants of each species at or near the resource three times a day (morning (6 : 00–8 : 00), afternoon (13 : 00–15 : 00) and evening (18 : 00–20 : 00)) for three consecutive days (i.e. nine observations) after removing the resource from the home box. Ants were considered to be using the resource when they were inside the nest vial, or within approximately 3 cm (1/4 of the length of the arena box) from the nest entrance, and when they were within approximately 3.4 cm from the food entrance (figure 1*c,d*). Because the ants could enter the vial of the nest but could not enter the food vial, we considered a slightly larger area around the entrance to the food vial to ensure that the resource use areas were of similar size for both resources (figure 1*c,d*). During each of the nine observations, we conducted four counts, one every 5 min, with each count lasting approximately 1 min. We summed these four counts to obtain a single value of the number of ants of each species using the resource in the arena for each of the nine observation periods in each of the eight replications. Some ants died over the course of the 3 days, therefore we counted the total number of ants alive of each species in each observation period to examine survival.

### (d) Statistical analysis of survival and resource use

To determine whether survival was impacted by the presence of a competitor and the number of resources available, we examined the change in the number of ants alive of each species over the course of our experiment (nine observations over 3 days). We ran a Cox proportional hazard regression model using the R package ‘coxme’ [42] in which the number of ants alive was the response variable and species (*L. humile* or *T. sessile*), type of resource(s) (food, nest, or both) and heterospecific presence/absence, i.e. trial type (experimental or control) were the explanatory variables. We included a three-way interaction term between species × type of resource(s) × trial type. For this and all following models, we performed *post hoc* tests for significant interaction terms with more than two levels using the emmeans() function in the R package ‘emmeans’ [43]. For interactions involving a continuous and categorical variable(s), we used the function emtrends(). We computed the marginal and conditional *R*^2^ using the R package ‘MuMIn’ [44].

To determine whether the use of resources changed over time, differed between the two species, resource types (food or nest), the number of resources (one or two), and was affected by the presence of a conspecific (experimental or control), we fit a generalized linear mixed model (GLMM) using a Poisson distribution. The number of workers at a resource was the response variable, and species, resource type (food or nest), number of resources (one or two), trial type (experiment or control) and time were the explanatory variables. We included group ID and starting date of the trial as random effects to account for the use of repeated measures of groups over the 3 days of the trial and any potential effects of seasonality (date). To account for different trends for the various effects, we further included two three-way interactions: trial type × resource × species and time × resource × species. The model was fit with maximum-likelihood (Laplace approximation) and implemented using the glmer() function from the ‘lme4' package [45]. The Anova() function from the ‘car’ package [46] was used for analysis of deviance to determine the confidence of our estimates using a Type II analysis of variance. All statistical analyses were conducted in R v. 4.0.2 [47].

### (e) Mechanisms of competition

To determine the behavioural mechanism underlying resource control, we observed aggressive interactions during the competition assays, noted which species arrived at the resource first and compared the exploratory behaviour of the two species. First, to determine whether aggressive behaviour had an effect on resource use, we counted the number of aggressive interactions at the resource(s) in the arena(s) during each of the nine observations. We recorded an aggressive interaction if we observed locking mandibles, biting, fighting, limb detachment, prolonged fighting and killing, as detailed in Neumann & Pinter-Wollman [33]. Because most aggressive interactions are brief, we used the total number of aggressive interactions (rather than the average) from the four 5 min samples in each of the nine observation periods, i.e. a total of 20 min in each observation period. We analysed the aggressive behaviour using a GLMM with a Poisson distribution, fit by maximum-likelihood (Laplace approximation), with the number of aggressive interactions as the response variable, resource type (food or nest), number of resources (one or two) and time as fixed effects, and group ID and date of trial as random effects. The model was implemented using the glmer() function from the ‘lme4' package [45] and the Anova() function from the ‘car’ package [46] to determine the confidence of our estimates using a Type II analysis of variance.

To determine whether the order of arrival at a resource, i.e. resource discovery, influenced resource use, we compared resource use, i.e. the number of workers at the resource(s) at the end of the three days, between trials in which *L. humile* arrived at the resource first and trials in which *T. sessile* arrived at the resource first. We used a GLMM with a Poisson distribution for count data, with final number of workers at a resource as the response variable, and species, number of resources (one or two), resource type (food or nest) and discoverer of resource (*L. humile* or *T. sessile* first at the resource) as fixed effects, and starting date of trial as a random effect. Group ID was not included as a random effect because this model only compares a single observation for each group—the last one.

Finally, to examine whether the two species differed in their exploratory behaviour, which might influence who discovered the resource first, we quantified the exploratory behaviour of 20 individual workers of each species that were not used in any of the other assays in an 8-armed maze with a novel spice at the end of each arm, as in Hui & Pinter-Wollman [35] and Page *et al*. [41]. To quantify exploratory behaviour, we recorded the total number of visits made by a worker to any arm of the maze over a 5 min period. Each worker was tested once because this assay provides an accurate and quick estimate of exploration that is repeatable over time and across situations, such as an open field test [41]. We then used a Wilcoxon sum rank test to compare the exploratory behaviour of *L. humile* and *T. sessile* workers.

## 3. Results

**Figure 2.**
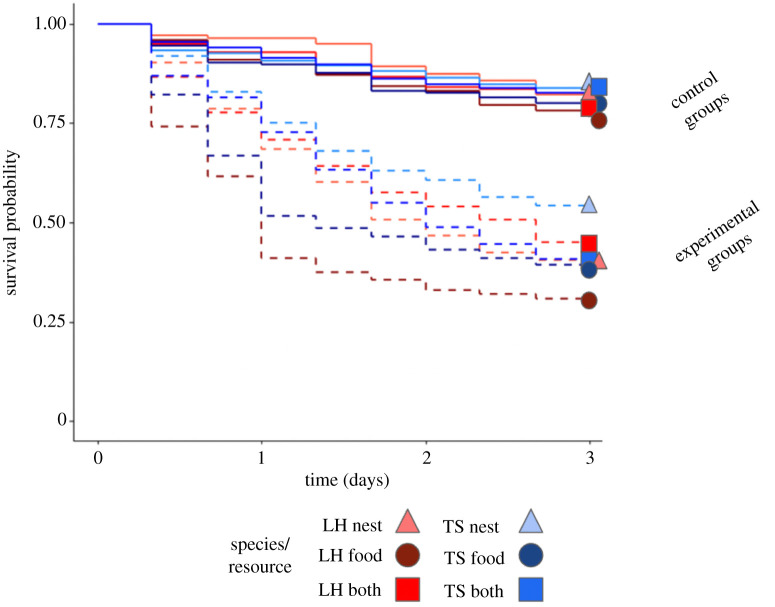
Survival of ants by species and study apparatus. Average proportion of ants surviving, relative to starting number (50 ants) at each of the nine observations over three days in experimental groups (dashed lines, *N* = 8) and control groups—when each species was alone in the apparatus (solid lines, *N* = 4). Blue lines are for *T. sessile* and red lines are for *L. humile*; different shades of blue and red and different shapes are for the different study apparatus (only nest: light blue/light red, triangles; only food: dark blue/dark red, circles; both resources presented: medium blue/medium red, squares). (Online version in colour.)

The number of ants alive decreased over time and the presence of a competitor reduced the survival of both species in all three treatments. For both species, workers were more likely to die in the presence of heterospecifics than in the absence of heterospecifics (Cox proportional hazard regression, trial type: *χ*^2^ = 397.80, *p* < 0.0001, *n* = 36; figure 2)*. Tapinoma* *sessile* had a greater survival rate than *L. humile* (*χ*^2^ = 9.97, *p* < 0.01, *n* = 36; figure 2). Specifically, there was a significant interaction between species and treatment (*χ*^2^ = 11.12, *p* < 0.01, *n* = 36; figure 2), with *T. sessile* having increased survival in the experimental nest treatment (blue versus red triangles in lower half of figure 2; *post hoc*: *z* ratio = 3.61, *p* = 0.016). For full output of this and all following models, see electronic supplementary materials.

**Figure 3.**
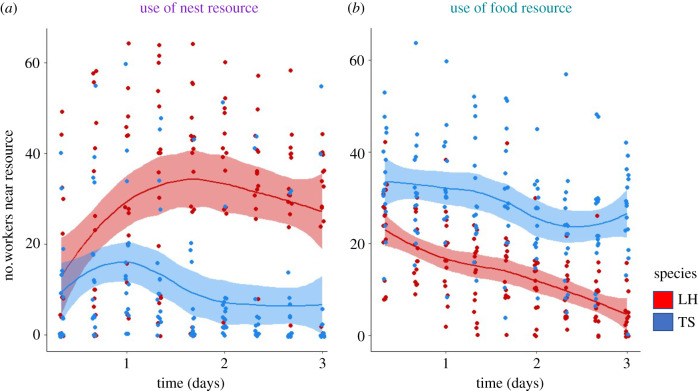
Use of resources over time. Number of *L. humile* workers (red) and *T. sessile* workers (blue) at the (*a*) nest and (*b*) food across the 3 days of the experiment (nine observations) (*N* = 8 replicates per treatment (food, nest or both)). Here and in following similar plots, data points are slightly jittered along the *x*-axis to improve visibility. Lines are the default loess fit in ggplot2 and shaded areas around the lines represent the confidence interval of the fit. (Online version in colour.)

Each species controlled a different resource (GLMM: resource type × species × trial type: *χ*^2^ = 913.34, *p* < 0.0001, *n* = 36; figure 3*a,b*). Specifically, in the experimental groups, *L. humile* had significantly more workers at nests (*post hoc*: *z* ratio = 33.58, *p* < 0.0001), while *T. sessile* had significantly more workers at food (*post hoc*: *z* ratio = −28.35, *p* < 0.0001), regardless of the number of resources present (GLMM: number of resources: *χ*^2^ = 0.44, *p* = 0.50, *n* = 36). Over time, the number of ants at each resource changed (GLMM: time: *χ*^2^ = 18.85, *p* < 0.001, *n* = 36) and this effect differed depending on the species and resource type (GLMM: time × species × resource type: *χ*^2^ = 213.23, *p* < 0.0001, *n* = 36; figure 3*a,b*). Specifically, the use of nests increased over time in *L. humile* (up-selected emtrend = 0.27) and decreased over time in *T. sessile* (down-selected emtrend = −0.06) and there was a significant difference between these trends (*post hoc*: *z* ratio = 10.44, *p* < 0.0001). Similarly, the use of food increased over time in *T. sessile* (up-selected emtrend = 0.16) and decreased over time in *L. humile* (down-selected emtrend = −0.11) and there was a significant difference between these trends (*post hoc*: *z* ratio = −12.37, *p* < 0.0001).

**Figure 4.**
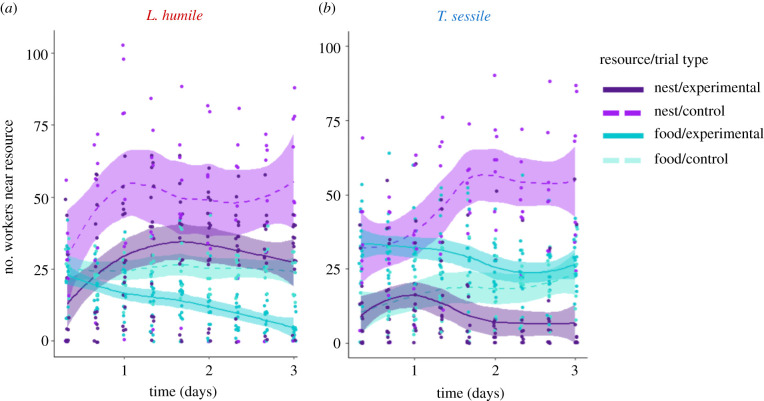
Effect of heterospecifics on resource use. Number of workers of *L. humile* (*a*) and *T. sessile* (*b*) at a resource (nest: purple; food: teal) for the different trial types (experimental: solid lines and darker colour shades; control: dashed lines and lighter colour shades). (Online version in colour.)

The presence of a heterospecific altered the use of resource(s) for both species. Overall, there were significantly more workers at a resource in the control trials, when no heterospecific was present (GLMM: trial type: *χ*^2^ = 18.61, *p* < 0.0001, *n* = 36; figure 4*a,b*), for both species (GLMM: trial type × species: *χ*^2^ = 2.03, *p* = 0.15, *n* = 36; electronic supplementary material, table S3a). However, species’ use of resources was impacted differently by the presence of a heterospecific (GLMM: trial type × resource type × species: *χ*^2^ = 913.34, *p* < 0.0001, *n* = 36). Specifically, in the presence of *T. sessile*, *L. humile* decreased their use of food (*post hoc*: *z* ratio = 6.78, *p* < 0.001; solid versus dashed teal lines in figure 4*a*), while in the presence of *L. humile*, *T. sessile* decreased their use of the nest (*post hoc*: *z* ratio = 9.09, *p* < 0.0001; solid versus dashed purple lines in figure 4*b*). Interestingly, there was a significant interaction between resource type and trial type (GLMM: trial type × resource type: *χ*^2^ = 179.63, *p* < 0.0001, *n* = 36). Specifically, there were significantly more workers at the nest than at the food in the control groups (*post hoc*: *z* ratio = −10.84, *p* < 0.001), suggesting that nests were the preferred resource for both species. The fixed effects of this model explained 52% of the variance (marginal *R*^2^) and the entire model explained 93% of the variance (conditional *R*^2^).

**Figure 5.**
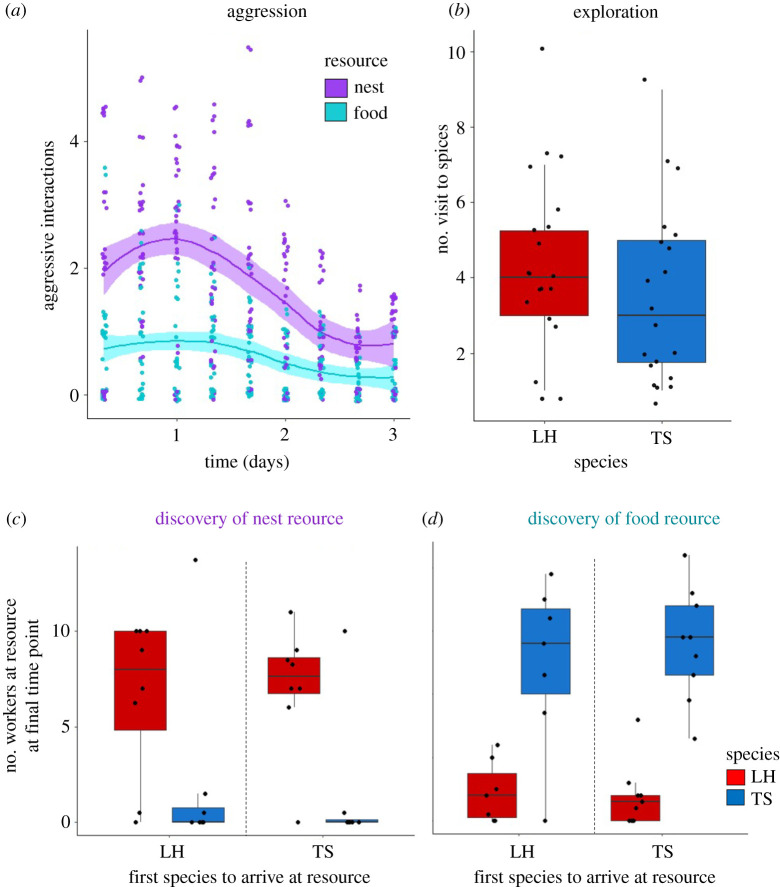
Mechanisms of resource use. (*a*) Number of aggressive interactions between *L. humile* and *T. sessile* at food (teal) and nests (purple) over time. (*b*) Exploratory behaviour of individual *L. humile* (red) and *T. sessile* (blue) workers (*N* = 20 each), quantified as the number of visits made to novel spices in an eight-armed maze. Number of workers at the nest (*c*) or food (*d*) on the last observation of the experiment, when either *L. humile* (red) or *T. sessile* (blue) discovered the resource first. (Online version in colour.)

The most likely behavioural mechanism driving the control of resources was aggression. There were significantly more aggressive interactions between *L. humile* and *T. sessile* at the nest than at the food (GLMM: resource type: *χ*^2^ = 372.14, *p* < 0.0001, *n* = 24; figure 5*a*) and this pattern was not impacted by the number of resources (GLMM: number of resources: *χ*^2^ = 0.022, *p* = 0.88, *n* = 24). Aggression decreased over time (GLMM: time: *χ*^2^ = 55.63, *p* < 0.0001, *n* = 24). The fixed effects of this model explained 59% of the variance (marginal *R*^2^) and the entire model explained 71% of the variance (conditional *R*^2^).

We did not find evidence supporting that the order of resource discovery or exploratory behaviour are likely mechanisms of competition. The number of workers at a resource at the end of the trial (final observation on day 3) was not significantly impacted by which species discovered the resource first (GLMM: species to discover resource first: *χ*^2^ = 1.44, *p* = 0.23, *n* = 24; figure 5*c,d*), nor was it impacted by resource type (GLMM: resource type: *χ*^2^ = 0.18, *p* = 0.67, *n* = 24; figure 5*c,d*) or the number of resources (GLMM: number of resources: *χ*^2^ = 2.31, *p* = 0.13, *n* = 24; figure 5*c,d*). The fixed effects of this model explained 11% of the variance (marginal *R*^2^) and the entire model explained 39% of the variance (conditional *R*^2^). Finally, we did not detect a significant difference in the exploratory behaviour of *L. humile* and *T. sessile* workers. Individuals from both species made a similar number of visits to the maze arms during the exploration trials (Wilcoxon rank sum test: *W* = 245, *p* = 0.22, *n* = 40; figure 5*b*).

## 4. Discussion

We found that interspecific competition affected survival, and that species differed in the resource they prioritized and in their influence on one another. When a competitor was present, survival was lower than when a competitor was absent (figure 2). The two species differed in their use of resources, with *L. humile* controlling nests (figure 3*a*) and *T. sessile* controlling food (figure 3*b*). The presence of *L. humile* significantly reduced the number of *T. sessile* at nests (figure 4*a*), while the presence of *T. sessile* significantly reduced the number of *L. humile* at food (figure 4*b*). Aggressive interactions likely drove the interspecific competition (figure 5*a*), rather than resource discovery (figure 5*c,d*) or exploratory behaviour (figure 5*b*).

Interspecific competition reduced survival overall. However, interestingly, *T. sessile* had higher survival rates than *L. humile* when competing over nests*.* In previous work, when colonies of 500 *L. humile* and *T. sessile* workers competed, *L. humile* had higher survival rates and they controlled both food and nests [31]. However, in our work with smaller groups of 50 workers, *T. sessile* fared better in terms of survival, which could have important implications for interspecific interactions during dispersal by propagules that can have as few as ten workers [36]. It is possible that small groups might not be able to control multiple resources simultaneously. Future work might consider whether the presence of a queen and brood influences competition and whether there is an optimal propagule size that allows both successful establishment when faced with interspecific competition and minimal investment by the colony in the number of dispersing individuals [48].

The patterns of worker distribution and aggression that we observed suggest that nests were preferred over food. Both species had more workers at nests than at food when the heterospecific was absent and just one resource was provided (figure 4*a,b*) and aggressive behaviour was more frequent near nests than near food (figure 5*a*). Furthermore, the ecologically dominant species, *L. humile* [28], controlled the nests when both species were present (figure 3*a*). The control of food by *T. sessile* could have resulted from ecological release [49], which occurred when the dominant *L. humile* expended workers to defend the nest, leaving *T. sessile* to forage freely at the food source. The release of *T. sessile* from competition at the food may have contributed to their increased survival when food was the only resource available (figure 2). Alternatively, it is possible that their higher survival rates allowed them to control the food source. Finally, because in urban environments that *L. humile* and *T. sessile* occupy, ants can exploit human food waste for sustenance [50], future work on competition in different ecological environments might reveal if the preference we observed for nests over food changes when food is a scarce resource.

Our results comparing the resource use of each species in the presence of the heterospecifics to resource use in the control trials, when heterospecifics are absent, suggest a potential allocation–control trade-off. Under the allocation–control trade-off, groups either allocate a small number of workers to all resources at the risk of not being able to control any single resource, or allocate many workers to a single resource and control it, at the risk of not obtaining enough of the other resource(s). The finding that both species reduce the use of one resource in experimental trials (*L. humile* reducing use of food and *T. sessile* reducing use of nests; figure 4*a,b*) suggests that both species were impacted by this trade-off. The focus of *L. humile* on nests and of *T. sessile* on food could be explained by competitive exclusion or by different physiological needs. Indeed, nutritional deficiencies of an ant colony can affect worker foraging decisions [51]. However, we show here that both a need for another resource simultaneously (like a nest) and the presence of another species may influence such decisions. Future work could examine how physiological constraints, such as starvation, further influence resource preferences.

Aggressive interactions between heterospecifics drove the competitive dynamics we observed more than difference between the two species in resource discovery or exploratory behaviour (figure 5). There were fewer aggressive encounters near the food, perhaps because workers at the food were hungry and sucrose deprivation decreases aggressive behaviour in *L. humile* [52], or because workers had just eaten and were not motivated to fight. These patterns of aggressive behaviour suggest that aggression may depend not only on species identity but also on the abundance of different resources in the environment.

The order of resource discovery and exploratory behaviour did not seem to have a significant impact on the interspecific competitive outcomes we observed. It is possible that discovering the resources was not a challenge to either species because the apparatus were small. Perhaps in a larger or more spatially convoluted setting we would have detected an effect of resource discovery order on resource control. Although competing species can differ in exploratory behaviour [24,53–56], we did not detect such a difference here. The lack of difference in exploratory behaviour could be explained by the high variation among individual workers in exploratory behaviour in both species. This variation can influence the ability of *L. humile* colonies to find a suitable nest [35] or food [41]. Thus, future work might consider how the behavioural composition of a group influences its competitive interactions [33,57]. The groups of workers in our study were competing in the absence of a queen or brood. It is possible that the presence of queens or brood would have resulted in different outcomes that could be examined in future work. For example, it is possible that the presence of queen and brood would lead to more aggressive behaviour by workers, especially when competing over shelter, because of their importance for reproductive success. It is also possible that interspecific interactions would persist for longer in the presence of brood and queen. For example, *T. sessile* workers might have continued to fight for the nest, rather than shift toward using the food (figure 3) if there were a queen and brood present to ensure their protection. Group composition is important for determining intergroup interactions and certain individuals (like a queen) may have a disproportionate impact on the dynamics and outcome of intergroup conflict [58,59]. Future research should explore whether there is a difference in resource use and competition when a queen (or queens) and brood are present.

To conclude, our findings suggest that identifying the causes and consequences of interspecific competition between social species requires consideration of environmental, behavioural and temporal effects. The result of a competitive scenario over one resource might not necessarily indicate how competitors will behave in the presence of other resources, other species, or on different temporal or spatial scales.

## Data Availability

Data and code can be found on Figshare: Data: https://figshare.com/articles/dataset/data_files_-_The_effect_of_resource_availability_on_interspecific_competition_between_a_native_and_an_invasive_ant/16786552. Code: https://figshare.com/articles/dataset/R_Code_for_analysis_-_The_effect_of_resource_availability_on_interspecific_competition_between_a_native_and_an_invasive_ant_/16786558.
